# Aseptic, Antiseptic and Septic Surgery

**Published:** 1888-10

**Authors:** J. McF. Gaston

**Affiliations:** Professor of Principles and Practice of Surgery


### Southern Medical Record.

A MONTHLY JOURNAL OF PRACTICAL MEDICINE.

☞All Communications must be addressed to R. C. WORD, M. D., Managing Editor.

"*Quicquid Præcipies Esto Brevis.*"

VOL. XVIII. ATLANTA, GA., OCTOBER, 1888. No. 10.

### ORIGINAL AND SELECTED ARTICLES.

### ASEPTIC, ANTISEPTIC AND SEPTIC SURGERY.

Address at the opening of the Session of the Southern Medical College, Atlanta, Georgia, 1888.

BY J. McF. GASTON, M. D.,
Professor of Principles and Practice of Surgery.

The advances in surgery within the past decade have been largely due to the adoption of measures of treatment based upon the germ theory of disease and the exclusion of the atmosphere, with its various impurities, from wounds.

It has been ascertained by microscopic investigation that many of the disorders of the organism are associated with, if not dependent upon, the presence of micro-organisms, and upon the hypothesis that these exert a causative influence upon inflammatory processes, the use of germicides is claimed by a large number of medical men, who occupy prominent positions in the profession, to exert a salutary influence. On the contrary, there are others of equal distinction who ignore entirely the operation of such an etiological factor, and contend that the presence of micro-organisms may be a concomitant or sequence of abnormal development in the tissues, which should not be taken into account, in employing therapeutic agencies in diseases.

The practical appliances in surgery, based upon the former assumption, have been so far successful as to sustain its advocates in its adoption for the treatment of all classes of injuries, and in the dressings of the wounds connected with most operative procedures. This view has taken hold of the profession to such an extent that an attempt has been made to impress the public with the idea that any other mode of proceeding should not be recognized by law, and departures from it should be considered as malpractice by the courts of justice. In other words that all who fail to observe the details of the so-called antiseptic surgery should be put under the ban of the profession, and that in medico-legal proceedings they should not be entitled to recognition, as fulfilling the indications of progressive surgery. It matters not in the estimation of these aggressive practitioners, that some of their colleagues who have had the same opportunities for studying this theory and similar occasions for observing the effects of its application, have not been able to discern its great practical benefits, and claim that their cases have been treated successfully without conforming to its requirements. A few, doubtless, who act upon their convictions, may be able to demonstrate by their results that the new regime is, to say the least, not necessary to success in surgery, and it behooves all to avoid the tendency to fall into line without proper examination of the merits of any method of treatment which happens to be popular.

There are some features of the observances inculcated by the new school philosophy which are troublesome and expensive, yet if they are essential to the proper treatment of a case, should not be neglected on this account. But if it is made to appear clearly that they are not requisite for a good result in a given case, or may be dispensed with advantageously, then it is certainly out of place to resort to an expensive and troublesome process when a more simple proceeding would serve a better purpose. Thus common sense would dictate the adoption of a course attended with the least embarrassment to the patient and the medical attendant, provided it proves equally satisfactory in its result.

The medical profession is not committed to accept the opinions of any class of practitioners solely upon their assertion of success with any special mode of treatment, as other modes of treatment may have been quite as efficacious in the experience of a different set of observers, and we are in the line of duty to weigh all the testimony available in regard to the innovations upon old and etablished practices before adopting any measure which has only the attraction of novelty or the weight of authority in its favor. Let us examine all things thoroughly, and after testing their virtues satisfactorily cling to those methods of treatment which are attended with the greatest efficiency.

There is a distinction between the normal and the physiological conditions of the nerves and vessels supplying a part in recent injuries to the organism by violence, as compared with the abnormal and pathological state resulting from inflammation or other structural disorders.

A proper discrimination between these, forms the basis of treatment in wounds at the different stages, and while a primary dressing is made with a view to the prevention of trouble beyond that of the traumation, the secondary measures may require a consideration of the modifications in the tissues and the consequent derangement of the general organization, including the vascular and absorbent systems. All changes in the organism of a secondary character depend upon an impression upon the nerve centres from the local injury, and thence the baleful influence is propagated through the efferent nerves and the ganglionic nerves throughout the entire organism, constituting what is characterized as constitutional disturbance. Topical means of treatment must be supplemented in this case by internal medication calculated to have a general effect upon the secretions. The action and reaction observed between traumatic lesions of a local character and the constitutional derangement involving the different organs and their functions, are as much within the province of surgery, as the treatment of the solutions of continuity in the tissues from violence that come under the care of the surgeon.

All the practical appliances of surgery may be appropriately classed under the headings of "Aseptic, Antiseptic and Septic Processes." The first embodies all those means which come under the class of prophylaxis, and looks to the avoidance of hurtful agencies, thus being assured that if a measure does no good it will do no harm. It is the highest type of conservatism to preserve the organs or tissues involved in an operation against any sort of mutilation or injurious impression from other sources. The procedure therefore which affords the best guarantee against detriment to the structure of a part subjected to surgical treatment, commends itself to the adoption of the practitioner under all the varied surroundings of the case. From whatever cause impairment or disability of the constituent elements of a part may ensue, whether in the action of a disease upon its tissues, or from the operation which may be undertaken, with the concomitant dressings, it should be put to the account of conservative surgery. The true aim and end of surgery being to preserve and not destroy a part which may be diseased or injured, all those processes of asepsis that look to its restoration are of the highest value to the surgeon. For illustration, it may be noted that a finger may be crushed and mangled in such a manner that amputation would be the quickest and easiest mode of procedure. Yet it would not be good surgery to sacrifice a part which by a proper appreciation of the recuperative powers of its tissues, and the timely adoption of an aseptic treatment should be saved. "Be sure you do no harm," is the great cardinal doctrine of aseptic surgery, and akin to this is the fundamental principle that "an ounce of prevention is worth a pound of cure" in guarding against trouble from any source.

It is a prudent policy to rely upon the *vis medicatrix naturæ* to a large extent in the management of recent traumatic accidents; and there is a masterly inactivity which avails much towards the restoration of structures that have been seriously injured by violence.

Some of the results of domestic management in the prompt readjustment of lacerated structures, and the application of simple remedies in wrapping up the part, call for the careful consideration of surgeons in making primary dressings of wounds. It is doubtful whether any improvement upon the blood plaster which is formed by the saturation of a loose cloth around a fresh wound, can be effected by any medicated application, and this soon forms a protection against the entrance of air, which serves as an aseptic dressing. The parts are in their normal healthy state with a predisposition to heal, and only require to be let alone; so that meddlesome surgery can only interfere with the cuperative process in the tissues, and we must play "hands off" in the management of this class of cases.

It is not proper to use disturbing measures either of a local or general nature, when the recuperative powers of an injured part are equal to the occasion, and practically our means of treatment are limited to the exclusion of the atmosphere from the wound. The province of the true asepsis in such cases is proper adjustment of the structures, and the prevention of anything calculated to interrupt the curative process.

A careful examination of a recent injury is of the utmost importance to detect foreign matters which may prove a hindrance to the restoration of the tissues by adhesive inflammation, and sufficient stress is not laid upon the fact that coagula of blood left in the bottom of even an incised wound may undergo decomposition and prove a source of serious trouble in the aseptic process. I am aware that the doctrine is held by some that a layer of blood clot over the bone and muscles after closing a stump in amputation of a limb is entirely innocent if not salutary. But my observation of the results in such cases, leaves not a shadow of a doubt as to the injurious consequences of leaving any accumulation, however small, within the flaps, where the skin is closed so as to prevent its escape, or hermetically sealed against entrance of air. To ensure a satisfactory issue in the application of aseptic dressings, it is essential that any exudation of blood or serum shall have an outlet, and he who neglects this precaution commits a grave error.

Extreme conservatism must be avoided in choosing the alternative between attempting to save a limb and sacrificing it, when the life of a patient is involved in the decision. With all our aseptic resources, it would be unwise to risk the consequences of an injury to the ankle joint in which the bones are crushed, the ligaments and tendons torn, and the nerves and arteries lacerated, though coaptation of the various structures might be to a great extent accomplished. The resort to immediate amputation under such circumstances is so imperative, that I should consider it rashness if not madness on the part of any surgeon who may undertake to save the foot by aseptic measures.

On one occasion, under similar conditions, when the patient insisted on an effort to save his foot, I withdrew from the case, and was subsequently recalled to amputate the leg at the point of election below the knee, whereas the operation could have been done previously at the ankle, without the grave constitutional symptoms that followed the injury.

With the lights before us, and the resources at our command for the preservation of the vital structures, the surgeon is warranted in undertaking to save limbs which under the old regime were necessarily lost. But it shows an utter lack of discretion to try to accomplish an impossibility by giving life to a dead member of the body.

Asepsis, as the derivation of the word implies, consists in opposing all contaminations from within and without a wound, and presupposes the vitality of the structures involved in the lesion, so that a part deprived of the supply of nerves and blood vessels, is "*ex-necesitate rei*," beyond the sphere of curative agencies, and its loss is a foregone conclusion. It is therefore good surgery to amputate such a member at the earliest practicable moment after the infliction of a destructive injury.

Barring this class, the most extensive traumatism may be amenable to proper diligence in the application of aseptic measures, and among them cleanliness is of prime importance. The idea conveyed by freedom from dirt or filth applies not alone to the absence of any foul extraneous matter from the wound, but its complete removal from the surroundings and the bedding of a patient as well as the apartment.

There are many cases in which medication is not requisite, and after the co-aptation of the tissues by suture or otherwise, a simple cotton dressing with a bandage suffices for the cure.

The water dressing for wounds so much in vogue formerly has been abandoned by most surgeons of late, owing to its many inconvencies in the repeated applications, and its positive hurtfulness in some instances by the extension of humidity to other portions of the body, leading to rheumatism, pneumonia, pleurisy, etc., more serious than the original injury

A fresh incised wound, either from accident or in the course of surgical operations, having the minute vascular supply in a favorable condition to secure union by adhesive inflammation between the approximated parts, only requires to be clean and free from coagula to heal without the interposition of medication. It is customary, however, to wash off such surfaces with a weak solution of carbolic acid before closing them, and to dust over the line of union with iodoform before enveloping the part with absorbent cotton, and this treatment is attended with satisfactory results. The washing out of the peritoneal cavity in laparotomy with a similar carbolic solution of a more dilute form, not exceeding five per cent., seems to be advantageous. But there is sufficient evidence from the practice of those who have employed a simple solution of chloride of sodium, for washing out the abdominal cavity, to rely upon its efficiency in such cases, and it is evidently innocuous; and it has been often resorted to for intravenous injections in cases of great vital depression from hemorrhage or other causes, with marked good results.

The possibility of leaving any portion of the fluid used as a wash in the abdominal cavity, and thus leading to its absorption, gives the chloride of sodium solution a decided preference over a carbolic solution, as the latter is liable to induce troubles of a nature which do not occur from the absorption of the former. The safest is therefore the best for general use.

There is a class of wounds attended with laceration and bruising of the adjacent structures, to which medicated dressings are essential for the prevetion of suppuration and the promotion of the recuperative process in the tissues of the part. In view of the impaired vitality of the organism under such circumstances, its capacity for restoration is lessened, and a stimulating embrocation becomes necessary to prevent the tendency to disorganization. As yet no septic process has been developed, but if the mangled structures are not assisted by a stimulant, a destructive decomposition may be expected in a brief period, and with a view to preserve the crippled capillaries and minute nerve fibres from death, no temporizing applications such as the canonized arnica and laudanum, or other mild applications are sufficient.

It has been found under such circumstances that all the requirements are satisfactorily met by a heroic treatment with a free application of spirits of turpentine and camphor, in the proportion of one drachm of the latter dissolved in one ounce of the former. This is poured into the lacerated wound and the part is enveloped in cotton saturated with it, having a bandage passed somewhat firmly around, so as to afford support to the bruised and weakened tissues. This dressing is repeated night and morning without washing the parts, and has in my experience proved to be the *ne plus ultra* as a surgical appliance in some of the gravest injuries of the hands and feet.

It is desirable in this class of injuries to prevent, as far as practicable, the suppurative process. After employing this dressing until there is a fair prospect of restoring the vitality of the structures and when the parts manifest increased sensitiveness, sweet oil is added to the liniment.

The camphorated spirits of turpentine being mixed with an equal quantity of olive oil forms a good application with absorbent cotton for continuous use, and it is rare that pus is formed in the course of the restoration of the tissues, even when the laceration has extended to the bone.

I am so impressed with the advantages of this mode of treatment that a typical case, recently under my observation, may be appropriately presented in illustration of its efficacy.

A white man had the left hand caught between the bumpers or deadwood of two railroad cars, crushing three of his fingers, the index, middle and ring fingers; the index being lacerated from the middle of the first phalanx to the middle of the third, opening the skin and subcutaneous tissues so as to expose the tendons of the flexor muscles, with detachment on either side from the bones; the middle having all the soft parts throughout its entire length mangled and the tissues torn away completely from the bone of the first phalanx beyond the articulation, so that all the surface of the bone was exposed and protruding; t e ring finger was also badly torn along its palmer aspect.

These required stitches for drawing the skin together at several points, and the middle finger was with much difficulty brought into shape by pressure.

Under the usual course of procedure, the first and second fingers would have been condemned and amputated, but my colleagues, Drs. G. G. Roy and P. E. Murray, concurring in the attempt to save them, I proceeded to bathe all the fingers with the camphorated turpentine, and wrapped each with cotton lint saturated with this liniment. Outside of this a layer of cotton was secured by a roller around each finger, and the entire hand was covered with cotton and wrapped up loosely, being suspended in a sling around the neck.

This dressing had been repeated daily under the supervision of Dr. Roy, who was in charge of the patient, and on the tenth day the wounds were found to be progressing favorably toward restoration in every respect without suppuration, except to a very limited extent, at the end of the middle finger. The nail which was left clinging to its matrix at the time of the first dressing had proved a source of irritation; whereas the end of the index finger from which the nail was detached with scissors at the original dressing, was healing kindly. My attempt to shut in the first phalanx of the index finger had not succeeded completely, and the point of the bone was exposed by the separation of the tissues, which I drew together by a strip of adhesive plaster. Some of the stitches had been previously removed, and those remaining were taken out on this occasion.

On the twentieth day this point of bone was nipped off, and the edges united by granulations, so that all the fingers healed by the end of a month, without such stiffening of any of their articulations as to impair their usefulness.

This is but one out of the many cases, with very similar conditions, which have been treated in this manner; and a number of my colleagues here are cognizant of all the facts connected with a large proportion of the successful results.

As there has rarely appeared an amount of pus to call for washing, most of the cases have been carried through the entire period of treatment without the use of water, and hence the aseptic properties of this combination are more strikingly exemplified.

Some progressive disciple of novelty may raise the point that there is nothing new in this, as camphor and turpentine are known as domestic remedies everywhere, and have been in use for bruises and wounds among the old women from time immemorial. Thus likewise, I have had occasion to refer to other aseptic measures used in domestic practice, which antedate Listerism by many decades, and it becomes the medical profession to recognize the fact that theoretically and practically cleanliness and exclusion of the air from wounds are not new doctrines. But it was necessary to have a systematic demonstration of this method of proceeding, and the hypothesis of the bacterial origin of suppuration and degeneration of structure after injuries, has furnished the occasion for much fine speculation by our medical philosophers, which may turn out to be well founded upon more thorough examination of the results. It is, however, for the present only to be regarded as a plausible explanation of the phenomena observed in wounds.

This brings us to the consideration of antiseptic applications in surgery, which differ widely from aseptic measures in their aim and results.

The indications for this class of remedies, being founded upon the anticipation or the existence of septic phenomena, lead some to a recognition of the microbic origin of such troubles, while others hold that the developments are attributable to other causes. The truth, no doubt, lies in avoiding the extremes on either side; and for practical purposes it is not requisite to decide as to the etiological factor in the septic process. It is a gratifying reflection for the medical philosopher and scientist to point out the separate germs of the various disintegrating processes involved in the aggravation of wounds, and to define the special microbe of distinct diseases, but the practitioner is not always prepared to accompany the bacteriologist in such investigations, nor does it devolve upon him to accept the conclusions from his investigations for the application of remedies in surgical cases.

After a somewhat careful research of what has been accomplished by the microscope in detecting the different forms of micrococci, spirilli, spores, etc., and noting the discrepancy between the observations of different experts in this department, I am still in doubt as to the role of bacteria in causing the degeneration of vital structures. That such micro-organisms, with fungi and ptomaines have a share in their development is evident, but whether the manifestations connected with their presence is a post hoc or propter hoc, has not been proven conclusively by the results of treatment. I know full well that this qualified incredulity will appear to those who are following in the wake of recent investigators as over cautious, but while questioning some of the tenets of this new faith, my practice conforms in the main to that of its apostles, and I hope to be able to give a reason for my departure from their procedures in so far as they may be hurtful to the patient.

To speak plainly, I am not following the dictation of any prophet in bacteriology, but shall exercise the right of private judgment in this as in all other matters involving professional observation and research. Having the courage of my conviction based upon investigation of this subject, I protest against a blind acquiescence in any and everything which may be styled antiseptic surgery, because forsooth it is backed up by respectable authority. There is much connected with the general observance of its doctrines which is commendable, and yet there are matters of detail entirely superfluous, and in some instances positively injurious to the patient. With all due respect to some of the prominent members of the profession who claim to be a privileged class in arrogating to themselves the matter of orthodoxy in professional faith and practice, touching antiseptic surgery, I must state that a highly intelligent portion of our colleagues do not accept the soft impeachment of heresy in differing from the standard set up for their guidance, nor admit their dictum that "I am more holy than thou."

The outline and embodiment of antisepsis is really confined to correcting a tendency to or a development of degeneration and disintegration of living structures resulting from contamination in any form. If we are able to discern the causative element and neutralize it by antiseptic measures of a constitutional or topical nature, this is of course the most rational procedure. But in the event of failure to identify the etiological factor, it then devolves upon the surgeon to combat the injurious effects of the vicious impression already made upon the organism and this is by far the most important province of antiseptic surgery. All that is done in anticipation of trouble belongs to the domain of asepsis, and yet the claim for antisepsis includes many measures supposed to be preventive of disorders which become sources of disturbance, so that instead of protecting the organization, they produce derangement and lead to septic developments. This class of agents comes under the division of septic surgery. It is now well-known that the imprudent use of carbolic acid locally has been followed by its absorption and a consequent derangement of the renal function. In like manner the employment of carbolic acid spray has proved os detrimental not only to the patient, but to the physician or surgeon, by inhalation of its noxious vapors, as to lead to its abandonment by most operators abroad, and it is to be hoped that ere long it will not be tolerated in this country, and only be heard of as "a school boy's tale, the wonder of an hour."

The evils growing out of the abuses of the bichloride of mercury as a germicide, are now so well-known to the profession generally throughout the length and breadth of the medical world, that I need not specify instances to any reader of current literature. Yet it is a daily practice with many members of our profession to employ solutions of this poisonous substance in operations involving the most delicate tissues of the body, with the facts staring them in the face that it is likely to be absorbed and produce serious inconvenience, if not fatal results.

Without any shadow of an excuse for a preference to the bichloride of mercury over other germicides less likely to prove hurtful to the patient, surgeons and gynecologists especially are vying with each other in the multiplicity of cases to which it is applied, and even physicians are contracting the mercuric mania, with the idea of killing microbes in the intestinal canal. The point is settled beyond controversy that it is a potent germicide, and the demonstration is completed by its efficiency in scabies, etc.; but if in the mad race after micro-organisms we do more damage to the structure in which they are discovered than the microbe is capable of doing if let alone then it behooves us to count the cost of such wild and reckless proceedings. I have a thorough comprehension of the temerity of making an issue against such odds as must be encountered in attacking the present popular rage in favor of surgical dressings with solutions of corrosive sublimate; but my voice will be heard and heeded by those who study the lesson of experience. Whenever the liability to disaster is so imminent as in the contact of the sublimate solutions with the absorbent surfaces or recent incisions, no caution short of interdiction meets the requirements of prudence; and except upon the cuticular investment of the body, there is no proper place for bichloride of mercury in antiseptic surgery.

It serves a good purpose for cleansing surfaces within the area of an operation and for disinfecting the hands of the operator and assistants, as it is not absorbed through the skin. But the use of sponges immersed in sublimate solutions, for application to raw surfaces in the course of operations, and the use of its solutions for dressings or washes to structures capable of absorption, should be ostracised by surgeons. With the lights before the profession in regard to the cases of poisoning resulting from even the most cautious employment of recognized dilutions of both carbolic acid and bichloride of mercury in surgical dressings, I am forcibly impressed with the conviction that they should be discarded in all operations upon normal structures. Many prominent practitioners think as I do, touching this septic contamination in what is claimed to be antiseptic treatment, and it is only requisite for them to take a bold attitude of opposition to such measures to effect the needed reform in surgical practice. If the deaths resulting from such mal-practice were traced to their true source,. instead of being attributed to the disease, the authors would incur a terrible responsibility.

There is a phase of septic development to which potent germicides are applicable, and when a local focus of contamination exists, whether it is associated with bacteria, fungi or ptomaines, the most vigorous use of antiseptic measures is demanded. It is evident that septic processes, which uncontrolled, must lead to septicæmia, are arrested in many instances by correctives of various kinds; and nothing which has been said should be construed as opposing a prompt resort to antiseptics and germicides, if indicated, in averting the progressive contamination of the general organization from local septic accumulations.

A normal healthy structure does not call for the application of germicides, but when the tissues have undergone a modification from some contamination, antiseptics are the sheet anchor of the surgeon.

Those prophylactics which may serve to prevent septic processes, and at the same time entail no injury upon the organization, are not amenable to the strictures made upon septic germicides; and it is held by some strenuous opponents of poisonous applications that innocuous agents may be employed as antiseptics with a more satisfactory result. Trusting that further investigation may lead to the adoption of means to arrest all septic processes without harm to the organism, I hope for such a triumph of antiseptic surgery.

